# The transverse four-chamber view for the assessment of atrial tissue deformation in the fetus

**DOI:** 10.1371/journal.pone.0199581

**Published:** 2018-07-02

**Authors:** Johannes Steinhard, Andreas Entenmann, Elise van der Valk, Ralf Schmitz, Jörg Heinig, Kai Thorsten Laser, Miriam Michel

**Affiliations:** 1 Center of Pediatric Cardiology and Congenital Heart Disease, Heart- and Diabetes Center North Rhine-Westphalia, Bad Oeynhausen, Ruhr University Bochum, Bochum, Germany; 2 Department of Pediatrics (Pediatrics I), Innsbruck Medical University, Innsbruck, Austria; 3 Department of Obstetrics and Gynecology, St. Franziskus Hospital, Münster, Germany; 4 Prenatal Medicine, Department of Obstetrics and Gynaecology, University of Münster, Münster, Germany; 5 Department of Pediatric Cardiology (Pediatrics III), Innsbruck Medical University, Innsbruck, Austria; University of Bern, University Hospital Bern, SWITZERLAND

## Abstract

**Aims:**

To determine if atrial tissue deformation (peak strain, PS) and time to peak strain (TTPS) can be assessed in the fetus, with identification of best echocardiographic plane.

**Materials and methods:**

Pulsed-wave tissue Doppler study of a longitudinal and a transverse four-chamber view (FCV) in each of 20 healthy fetuses. Determination of PS and TTPS in regions of interest (ROI), *viz*., lateral walls of the right and left atria (RA, LA); comparison of values depending on section plane, with results-based discussion of the physiology of fetal atrial deformation and of possible clinical uses.

**Results:**

PS and TTPS could be determined on transverse FCV in 91% of subjects and in 61% on longitudinal FCV. Transverse PS and TTPS were significantly higher than longitudinal (p = 0.0001). Transverse PS was significantly higher in RA than in LA (26.9% *vs*. 17.3%, p = 0.034), and transverse TTPS was significantly shorter in RA than in LA (p = 0.034).

**Conclusion:**

Atrial radial PS and TTPS determinations are possible in the fetus. The transverse FCV is best suited for these. The highest PS values and shortest TTPS values are found in ROI representing the RA. Our findings may contribute to detailed intrauterine assessment of atrial and ventricular myocardial function.

## Introduction

The fetus has specific myocardial properties and a physiologically high heart rate (HR). Immature diastolic ventricular function entrains myocardial deformation, thus depending on diastolic ventricular filling via atrial contraction. Increased HR, not ejection fraction, physiologically compensates for hemodynamic impairment. Diastolic and systolic dysfunction results, manifest as ascites, pleural pericardial effusions, or death. Early, precise detection of origin and amount of ventricular myocardial systolic and diastolic dysfunction is essential for therapy. Fetal *ventricular* performance is mainly quantified by conventional M-mode echocardiography [1]. Determination of fetal *atrial* function classically comprises analysis of atrial rhythm and atrioventricular (AV) interaction, requiring simultaneous pulsed-wave Doppler interrogation of mitral-valve inflow and aortic outflow; of flow in superior vena cava and ascending aorta; of flow in pulmonary artery and pulmonary vein; or simultaneous M-mode echocardiography of both ventricle and atrium [2, 3]. These techniques require special training, optimal fetal position, and are time-consuming. This impedes early detection of impaired myocardial performance or dysrhythmia.

Sophisticated analyses via tissue-deformation echocardiography allow quantification of myocardial function [4–8]. From *ventricle* these techniques have moved to *atrium* in adults, children [9], and fetuses, mainly focusing on the left atrium (LA) with respect to tissue *velocity*, assessing rhythm and conduction [4, 5, 10]. We know of only one published study of *atrial* peak strain (PS) using speckle tracking echocardiography (STE) to identify preserved *atrial* longitudinal PS in fetuses [11]. As STE uses a lower frame rate (FR) than tissue Doppler (TD), TD yields higher temporal resolution. We aimed to evaluate if echocardiography permits quantification of fetal *atrial* PS and time to peak strain (TTPS) using TD and to determine the best echocardiographic approach to the atrial walls [6]. On the basis of these results, we discuss the physiology of fetal atrial deformation.

## Material and methods

We retrospectively analysed 75 four-chamber views (FCV) of hearts of fetuses whose mothers presented to the Division of Prenatal Medicine, Department of Obstetrics and Gynaecology, University of Münster, Germany, over the study period (1y). Inclusion criteria were appropriate image quality, normal fetal cardiac structure and function, and normal fetal sinus rhythm: 20 fetuses (each with a longitudinal and a transverse FCV) were studied. The mothers were referred either for second- or third-trimester screening or specifically for fetal echocardiography due to a family history of congenital heart disease. Each fetus underwent examination according to International Society of Ultrasound in Obstetrics and Gynaecology guidelines. Loops were recorded using a multi-frequency 5 MHz sector scanner (PST-50AT, Aplio 80 research ultrasound system, Toshiba Medical Systems Europe, Zoetermeer, The Netherlands), with pulse repetition frequency lowered and wall filter and receiver gain reduced to obtain tissue motion signals. Atria needed to be displayed *in toto* from the AV valves to the level of the atrial roof. The maximum accepted angle of insonation was at 20° to the interventricular septum in longitudinal FCV or to the AV plane in transverse FCV, and we attempted to keep FR at >130 frames per second (FPS).

### Off-line analysis

**Fig 1:**
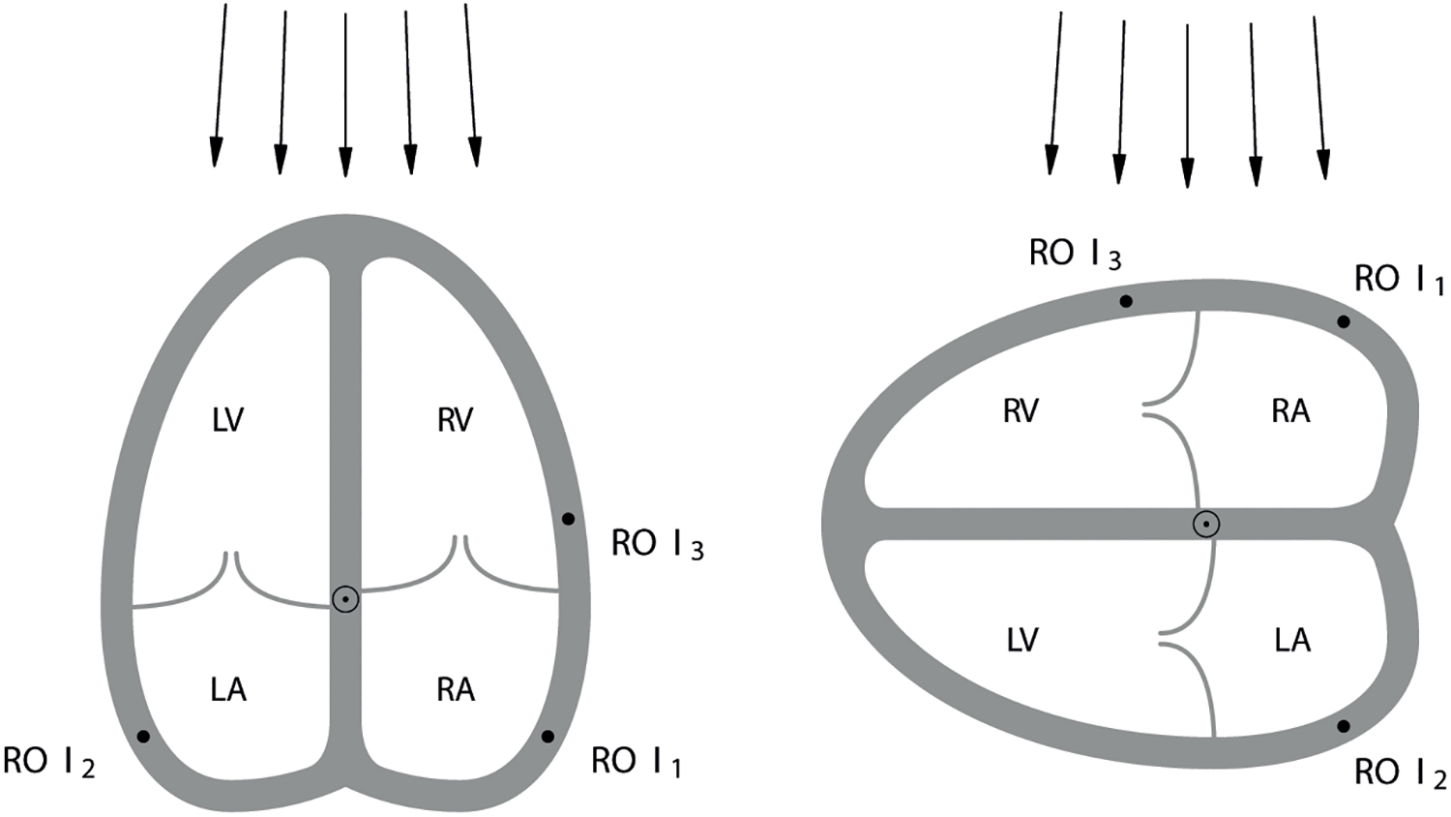
Schema of a longitudinal (left) and a transverse (right) four chamber view of the heart, showing ROI placement in the right atrium (*ROI 1*), left atrium (*ROI 2*), and right ventricle (reference ROI; *ROI 3*). Since in the longitudinal view the ultrasound beam is directed apico-basally, atrial structures are distant and atrial lateral walls are met tangentially. In the transverse view the ultrasound beam meets the lateral atrial walls perpendicularly. ROI, region of interest; RA, right atrium, LA, left atrium; RV, right ventricle; LV, left ventricle.

Pulsed wave TD raw data loops were processed (TD Imaging Quantification software, version 1.7, TDIQ, Toshiba Medical Systems Europe). PS was quantified (AxioVision software LE, version 4.4.0.0, Carl Zeiss Vision, Jena, Germany). 2-mm circular regions of interest (ROI) were manually placed at the level of the lateral atrial wall (ROI 1 = right atrium [RA], ROI 2 = LA) (Fig 1).

There was no automated feature tracking. Closure of the aortic valve was determined. In order to define a single heart cycle and to check TD data quality we analysed right ventricular myocardium velocity at the level of the tricuspid valve lateral annulus (ROI 3) [12]. A centre of contraction was set at the intersection of interatrial and interventricular septa at the level of the AV valves. PS (%) in the direction towards this centre of contraction (radial PS; positive, lengthening; negative, shortening), and TTPS values were determined. PS and TTPS from a transverse FCV (lateral window) were compared with those from a longitudinal FCV (apical window). The loop was considered usable if the clear definable PS was ≥5%.

### Curve interpretation

**Fig 2:**
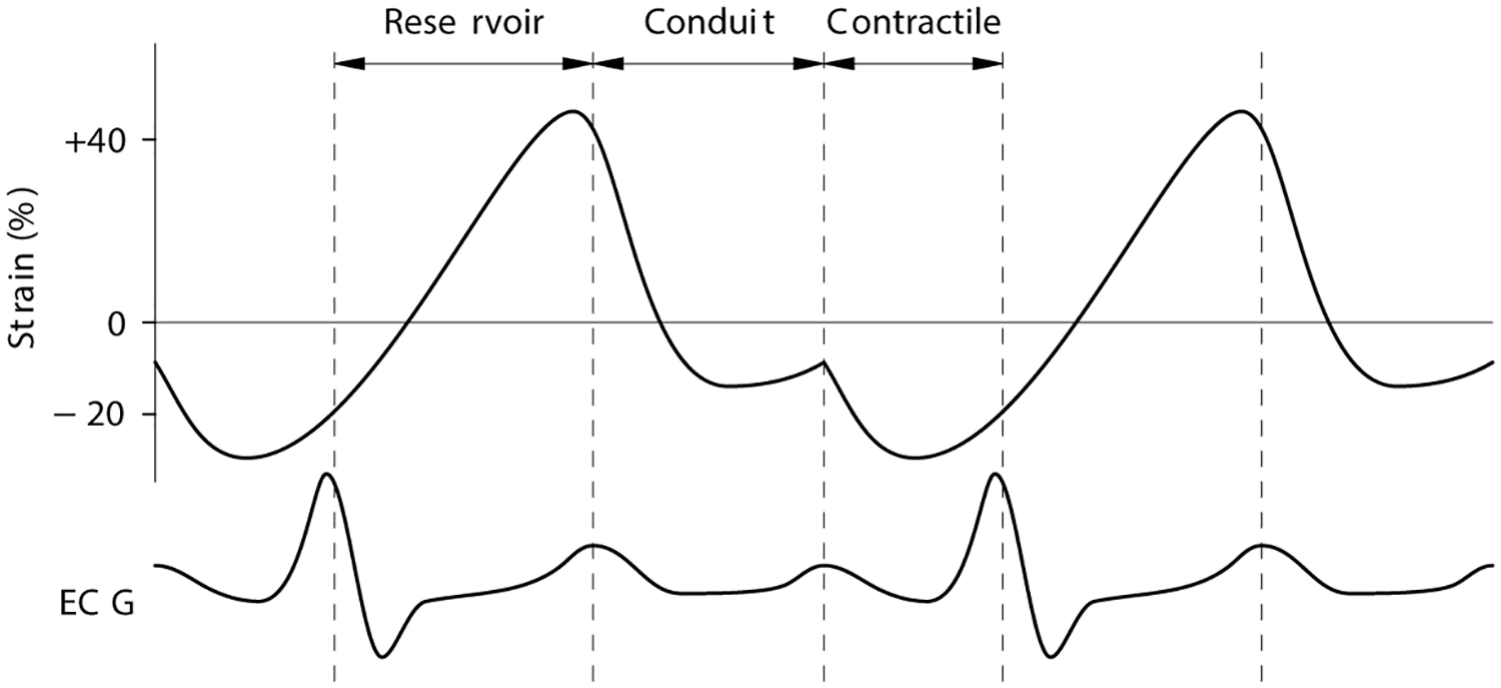
Triphasic atrial longitudinal strain dynamics during the cardiac cycle. ECG, electrocardiogram.

TD-derived strain of the atrium (LA>RA) reflects three phases, closely following left ventricular (LV) dynamics during the cardiac cycle (Fig 2, S1 Fig):

- LA *reservoir* phase; during LV systole and reflecting LA suction force (influenced by movement of the mitral annulus toward the cardiac apex), the LA collects pulmonary venous flow. Longitudinally, the LA stretches and strain increases, peaking as atrial filling ends. Radially, the LA wall thickens (strain nadir).
- LA *conduit* phase; the LA empties blood into the LV during early diastole. Longitudinal strain falls and plateaus, corresponding to LV-wall diastasis, and radial strain increases.
- LA *active contractile* phase; the atrial wall shortens longitudinally and thickens radially during LA contraction, with longitudinal strain decrease and radial strain increase.

In many fetal heart strain curves the peaks following conduit and active contractile phases are not optimally depicted and thus hard to identify. We thus focused on atrial reservoir-phase PS, in correlation with atrial longitudinal stretching (positive values, peak strain) and radial thickening (negative values, strain nadir) [5, 9–11, 13].

### Statistics

Data are expressed as median and interquartile range. Non-parametric Mann-Whitney-testing was used to compare PS and TTPS originating from the two different atrial ROI and PS and TTPS originating from the two different echocardiographic approaches. p-values ≤0.05 were considered significant. Statistical analysis was done using SPSS 20.0 (SPSS, Chicago, IL).

### Ethical considerations

All investigation was conducted according to the principles expressed in the Declaration of Helsinki. The study was approved by the institutional review board of the University of Münster, Germany. Informed consent for study participation was obtained retrospectively from each mother.

## Results

Forty FCV qualified for TD assessment. For general and echocardiographic characteristics of the study population see Table 1.

**Table 1:** General and echocardiographic characteristics of the study population.

	Longitudinal	Transverse
**GA [wks]**	23:3 (20:2 to 29:5)	23:3 (21:3 to 28:5)
**HR [1/min]**	144 (135 to 151)	148 (141 to 151)
**FPS [1/s]**	140 (132 to 160)	135 (123 to 156)

Data are median (interquartile range). Note that differences between longitudinal and transverse data were not significant. Longitudinal, longitudinal four chamber view; Transverse, transverse four chamber view; GA, gestational age; wks, weeks; HR, heart rate; FPS, frames per second.

**Fig 3:**
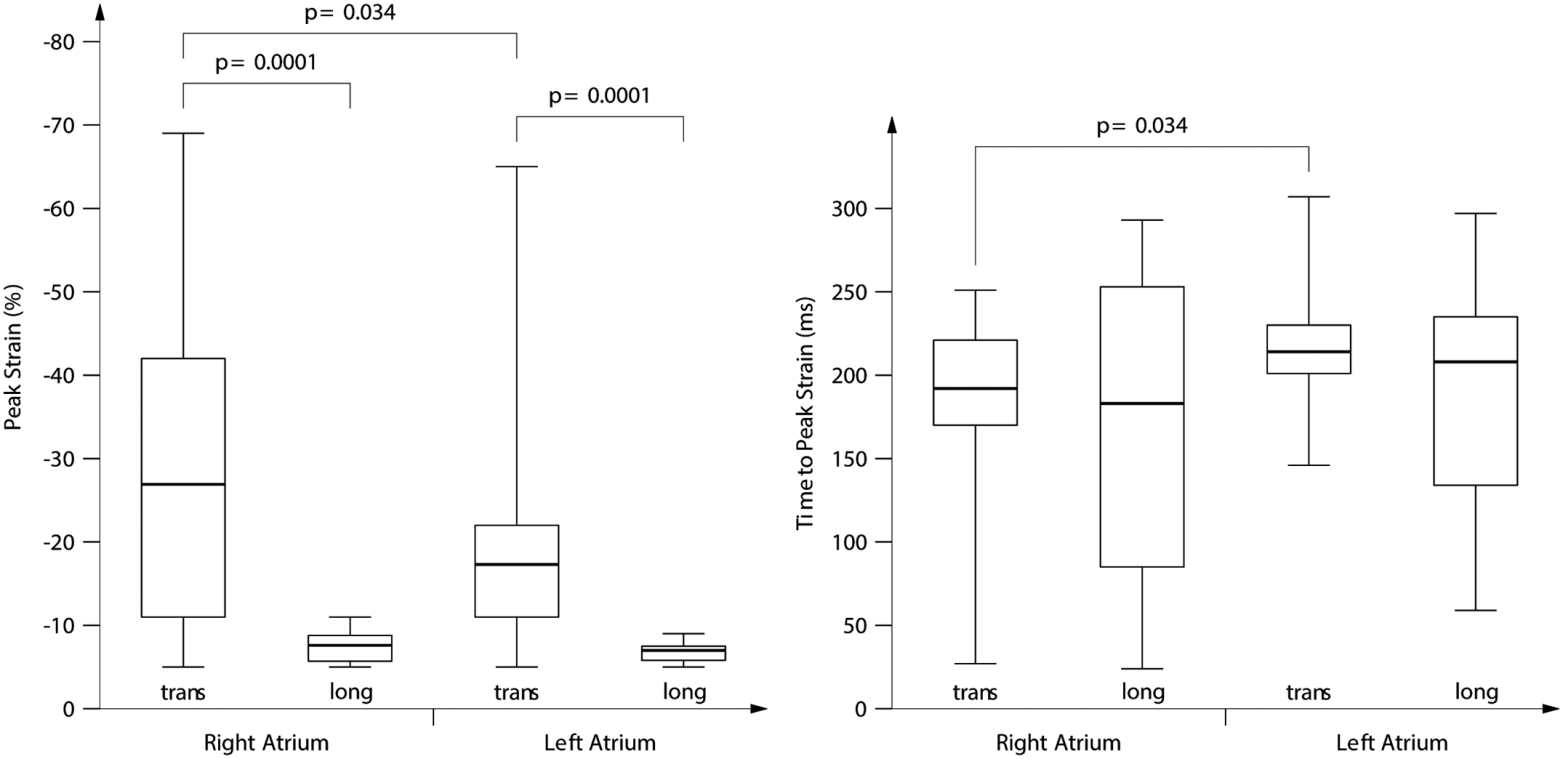
Absolute peak strain (left) and time to peak strain (TTPS) (right) for right (RA) (*ROI 1*) or left atrium (LA) (*ROI 2*) in the transverse (trans) or longitudinal (long) four chamber view. Note that for the sake of visual comparison, numbers for strain are depicted as values so that numbers for both longitudinal and radial strain are “positive”; normally, radial strain (transverse four chamber view) would be negative. The boxplots indicate the median (interquartile range), the whiskers reach from minimum to maximum. The p-value indicates the statistically significant difference between results.

Quantification of PS and TTPS was feasible in transverse FCV in 91%, and in longitudinal FCV in 62%. In both ROI, PS in transverse FCV was significantly higher than in longitudinal FCV (p = 0.0001). In transverse FCV, PS originating from ROI 1 (*viz*., RA) was significantly higher than PS originating from ROI 2 (*viz*., LA) (p = 0.034). Longitudinal PS did not significantly differ between the atria (Table 2, Fig 3).

**Table 2:** Radial and longitudinal peak strain of regions of interest both ROI reflecting the atria.

	PS [%] ROI 1	PS [%] ROI 2	p
**L**	7.6 (5;11)	7 (5;9)	0.369
**T**	-26.9 [5;69)	-17.3 (5;65)	0.034

Median (minimum; maximum) radial and longitudinal peak strain (PS) [%] of regions of interest (ROI) *ROI 1* (right atrium) and *ROI 2* (left atrium).

TTPS in transverse FCV was significantly longer in ROI 2 than in ROI 1 (Table 3, Fig 3).

**Table 3:** Radial and longitudinal time to peak strain of regions of interest both ROI reflecting the atria.

	TTPS [ms] ROI 1	TTPS [ms] ROI 2	p
**L**	183 (23;293)	208 (58;297)	0.241
**T**	192 (27;250)	214 (146;306)	0.034

Median (minimum; maximum) radial and longitudinal time to peak strain (TTPS) [ms] of regions of interest (ROI) *ROI 1* (right atrium) and *ROI 2* (left atrium).

## Discussion

In the healthy fetus TD-derived atrial radial reservoir PS and TTPS can be evaluated, with best detection of PS if assessed in transverse FCV and with longest TTPS in ROI-2 (LA).

**Fig 4:**
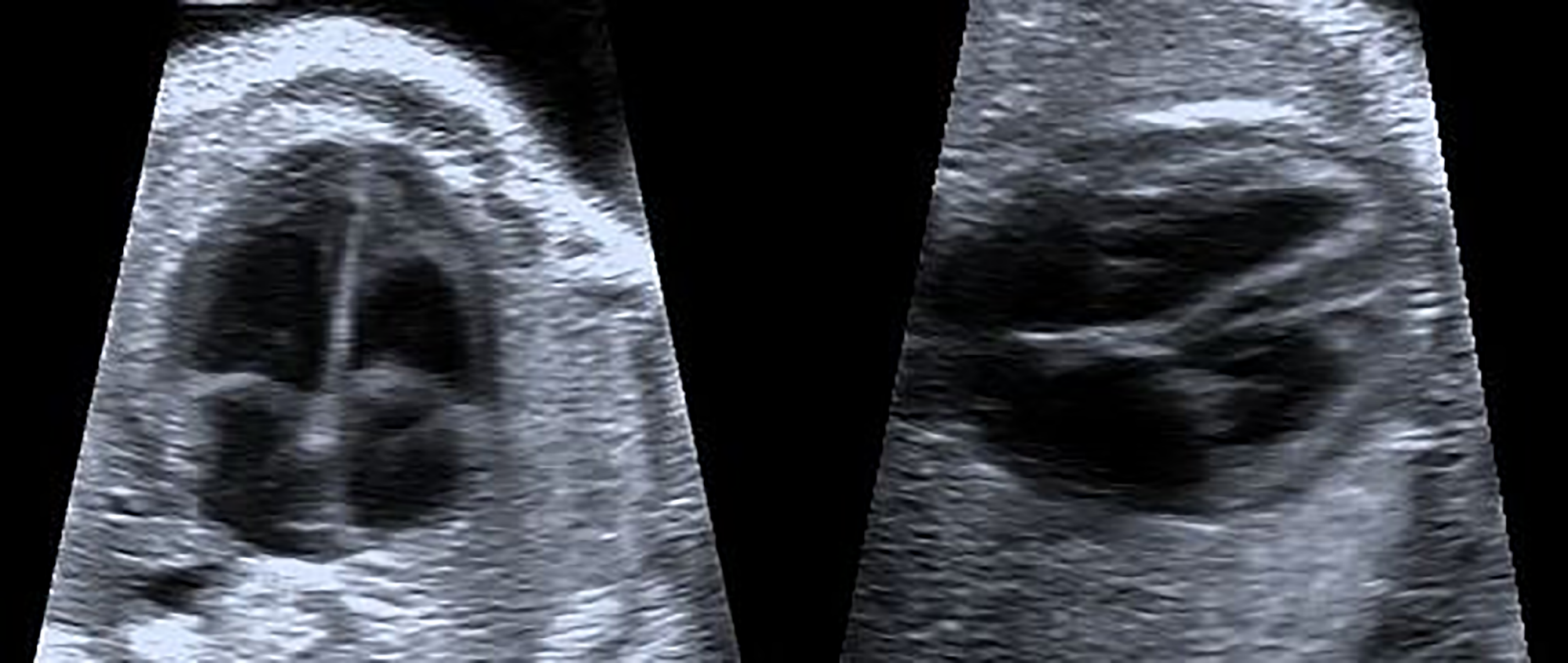
Example of a four chamber view of a fetal heart in longitudinal or apical (left) and transverse (right) section. Note that the interventricular septum and especially the thin interatrial septum are displayed best in the “in plane-view” of the transverse view where the ultrasound waves meet those structures perpendicularly.

In adults, reservoir PS correlates positively with LV filling pressure in LV systolic dysfunction [14]; predicts LV late remodeling after acute coronary syndrome [15]; falls immediately after cardioversion, reflecting atrial stunning [16]; falls in mitral or aortic valve disease [17, 18]; and correlates negatively with levels of brain natriuretic peptide n-terminal prohormone [19]. Atrial PS in children has been reported rarely, comparing LA and RA reservoir PS in patients after surgical or interventional closure of an atrial septal defect [9]. The only study of fetal atrial PS known to us found *global* atrial PS in fetuses with placental dysfunction to be preserved; it used STE [11], which is not yet fully customized for fetal echocardiography [6]. Ours is the first *TD* study examining the feasibility of *regional* atrial PS in healthy fetuses, focusing on reservoir PS (or “PS during ventricular systole” [11]), but with emphasis on *radial* instead of *longitudinal* PS, additionally comparing PS from two different views–transverse and longitudinal–and analysing TTPS. That we best detected radial deformation in the lateral walls of the fetal atrium assessed in transverse FCV reflects the nature of ultrasound: Waves perpendicularly impinging on a surface and reflected perpendicularly (*cf*. Snell`s law) provide optimum echoes with best spatial resolution [20]. The importance of optimum echoes in assessment of tiny cardiac structures in the fetus via “in-plane view” applies not only to ventricular septal defects [21] but also to the thin and smooth atrial walls, which if assessed in longitudinal FCV lie almost tangentially to the ultrasound beam (Fig 4).

In apical FCV the atria are far from beam origin, yielding lateral resolution less than that in transverse FCV (with the atria nearer the transducer [22]). With *longitudinal* FCV, *longitudinal* PS of lateral atrial walls could readily be measured, as could *radial* velocity of the lateral walls, consonant with examination of global instead of regional deformation and using apical, basal, and slightly angled FCV [11]. Amplitude of atrial mechanical deformation is expected to be higher longitudinally than radially [23]. However, beyond optimum perpendicular echocardiography, fetal atrial anatomy and tissue properties may also facilitate radial strain quantification. While the lateral and anterior atrial walls are free, the cranial and dorsal walls are attached to the adjacent systemic and pulmonary veins. In this “fixed” region, tissue is less contractile than in the “free” region, resulting in PS values higher in atrial septum and lateral RA and LA than elsewhere in the atrial roof as well as in easily detected radial tissue movement in lateral regions [24, 25].

With transverse FCV, we saw the highest PS values in ROI 1, representing the *RA*. This corresponds to earlier findings [11]. Contributory might be both placement of the ultrasound beam closer to RA than to LA structures and RA structures themselves (pectinate muscles of the RA appendage and the terminal crest). Within these thickened regions, most myocytes lie parallel to the long axis of the muscle bundles, while in LA wall myofiber orientation varies with depth [25]. Filling pressure is lower and compliance higher in RA than in LA, which allows more absolute movement of RA than of LA tissue [26]. Due to reduced atrial compliance, LA tissue reflects passive “manipulation” more intensely than RA tissue, yielding more distorted strain curves with several smaller peaks. As fetal cardiac tissue is stiffer than paediatric and adult, atrial compliance may be less than postnatally. On the other hand, reduced ventricular relaxation in the fetus might exaggerate the impact of AV valve movement on atrial mechanics. Thus, reduced ventricular myocardial compliance is important in analysis of fetal tissue movement, as described in adult hearts [27].

Fetal physiological right-to-left shunting via the foramen ovale may lead to greater movement of RA tissue than LA tissue, implying good measurability of PS in the RA, while the movement of the septum primum into the LA could impede LA assessment and simultaneously alter LA mechanics.

Our second major finding, that TTPS duration varies, and is greater in ROI 2 (LA), accords with findings of electrophysiological studies in the postnatal heart. Electrical impulses spread from upper RA via lateral atrial wall towards tricuspid valve and via atrial roof to atrial septum and LA, leading to physiologic intraatrial conduction “delay” of electrical signal and mechanical tissue action [4]. The lack of variation in TTPS in longitudinal FCV underscores the superiority of transverse FCV in displaying fetal atrial structures and mechanics. In our study HR did not differ significantly between longitudinal and transverse recordings and thus should not have influenced TTPS.

### Clinical implications

In the fetus, the transverse FCV facilitates the clinical application of quantitative analyses of atrial lateral wall mechanics. Direct assessment of atrial PS during the *reservoir* phase may help evaluate diastolic and systolic atrial and ventricular myocardial function. Additional analysis of fetal diastolic myocardial function–already constrained by tissue stiffness (especially of the ventricular myocardium) and by high HR–would be particularly helpful.

If confirmed by intra- and interobserver studies and after having established gestational age-adjusted reference values on larger sample sizes, focusing on PS during the atrial *contractile* phase may permit automatic detection of “suspect” atrial events that indicate atrial dysrhythmia, prompting detailed prenatal cardiologic evaluation and postnatal cardiologic referral.

As the technique is easy to apply in terms of equipment, investment, and learning curve, it might in daily practice facilitate detection of *fetal dysrhythmia* of supraventricular origin, or of *tachycardia with AV reentry mechanism*. This technique might also help precisely determine *AV conduction time*, even if which TTP best correlates with definite atrial movement must still be proven [4]. Here the longest TTP, which in sinus rhythm usually should be assessed at the level of the lateral mitral-valve annulus, perhaps with a mean of all atrially assessed TTP, might be appropriate. Measurement of *total atrial conduction time*, corresponding with p-wave duration in electrocardiograms, might be another option. In adults this is used to predict early recurrence of persistent atrial fibrillation after successful electrical cardioversion [28].

### Limitations

Varying anatomy; in addition, tiny structures might especially in the longitudinal FCV simply “move away from the ROI”, thereby yielding lower longitudinal than transverse PS values. In the transverse approach, motion of other atrial segments might have been detected, contributing to higher values. This might also underlie increased variability of transverse-approach values, a point to be assessed by studies of reproducibility and of intra- and interobserver variability, which could not be performed in our study, due to its retrospective nature. The limited temporal resolution of the TD technique is the main obstacle to the exact evaluation and comparison of atrial PS. In our study the median FR of 139 FPS dependably allowed detection of a peak value no more frequently than every 7.2 ms. Thus, we cannot with certainty exclude that some peaks were missed [4, 5, 11, 29]. Especially in the fetal heart, with a physiologically high HR, a FR of about 300 FPS would be optimal. This is especially true for rhythm assessment, where PS must be evaluated during the atrial contraction phase: The peak or nadir is relatively indistinct, and thus difficult to depict and to differentiate from the peak during conduit phase. An optimal cut-off of a minimum value accepted as “real” PS should therefore be defined. Our arbitrary definition of a minimum PS of 5% and the ROI size of 2 mm ensured suppression of “noise” peaks while concomitantly guaranteeing that major peaks would not be missed [5, 29]. Examination of several ROI within each atrial wall would be interesting but technically challenging [13].

In transverse FCV, we did not strictly target the same side of the heart, which might have influenced lateral resolution as well as PS and TTPS. However, as the difference in PS between transverse and longitudinal FCV was substantial and can be explained by the physics of ultrasound, we considered those limitations unlikely to affect our main findings substantially. Besides further intra- and interobserver studies and besides the establishment of gestational age-adjusted reference values on a larger sample size, comparisons with conventional ultrasound techniques and fetal cardiac magnetic resonance imaging are essential for adoption of assessment of fetal atrial deformation into clinical medicine.

### Conclusion

TD-derived atrial *radial reservoir* PS and TTPS can be assessed in the normal fetal heart. *Transverse* FCV yielded highest values for PS in ROI 1 (RA) and for TTPS in ROI 2 (LA). However, various limitations of parametric imaging of the fetal atrium still must be quantified in detail. Once tested by intra- and interobserver studies, *atrial PS* measurement may allow early and precise evaluation of fetal ventricular myocardial dysfunction and of rhythm and conduction abnormalities, thereby permitting physiologically based therapy.

## Supporting information

S1 Dataset Patient information and data of echocardiographic atrial measurements. (XLS)

S1 Fig Sample curves of one heart cycle of peak longitudinal strain. The colours of the graphs correspond to the respective regions of interest (ROI): red, *ROI 1* (right atrium); green, *ROI 2* (left atrium).
(EPS)

## Abbreviations

% (percent): peak strain
° (degrees): angle of insonation
**AV**: atrioventricular
**FCV**: four-chamber view
**FPS**: frames per second
**FR**: frame rate
**HR**: heart rate
**LA**: left atrium
**LV**: left ventricle
mm (millimeters): size of region of interest
ms (milliseconds): time to peak strain
**PS**: peak strain
**RA**: right atrium
**ROI**: region of interest
**STE**: speckle tracking echocardiography
**TD**: tissue Doppler
**TTPS**: time to peak strain
